# Effects of mirror therapy combined with theta burst stimulation on motor recovery of upper limbs after stroke: a randomized controlled study

**DOI:** 10.3389/fneur.2025.1548703

**Published:** 2025-07-11

**Authors:** Jing Zhou, Mo Chen, LuJie Dong, CaiXia Zheng, Jiang Xu, YangPu Zhang, YaLi Liu

**Affiliations:** ^1^Department of Rehabilitation Medicine, Tongji Hospital, Tongji Medical College, Huazhong University of Science and Technology, Wuhan, China; ^2^Department of Rehabilitiation, Hubei Provincial Hospital of Integrated Chinese & Western Medicine, Wuhan, China

**Keywords:** stroke, mirror therapy, theta burst stimulation, upper limb function, motor evoked potential

## Abstract

**Objective:**

This study aimed to explore the impacts of intermittent Theta burst stimulation (iTBS) and mirror image therapy (MT), both used separately and in combination with iTBS, on upper limb (UL) motor function, activities of daily living (ADL), and cortical excitability during the subacute phase of stroke.

**Design:**

Randomized controlled study.

**Setting:**

Inpatient rehabilitation centers of Tongji Hospital and Hubei Provincial Hospital of Integrated Chinese & Western Medicine.

**Participants:**

Seventy-one patients with upper limb (UL) disability.

**Interventions:**

Patients were randomly assigned to four groups. Three treatment groups received intermittent theta burst stimulation (iTBS), mirror therapy (MT), or a combination of both, in addition to routine rehabilitation. Therapy sessions were conducted five days per week for two weeks (10 working days).

**Main measures:**

The assessments encompassed the National Institutes of Health Stroke Scale (NIHSS), upper limb Fugl-Meyer assessment (UL-FMA), modified Barthel index (MBI), Stroke-specific quality of life scale (SS-QOL), resting motor threshold (RMT), and motor evoked potential (MEP).

**Results:**

The combined treatment group showed significant improvements in UL-Fugl-Meyer Assessment (UL-FMA) scores compared with the control and MT groups (*p* < 0.05). Significant differences in Modified Barthel Index (MBI) and Stroke-Specific Quality of Life Scale (SS-QOL) scores were observed among the four groups (*p* < 0.05). On the contralesional side, the iTBS group demonstrated increased resting motor threshold (RMT), prolonged motor evoked potential (MEP) latency, and reduced MEP amplitude. In contrast, the MT group showed decreased RMT and MEP latency, along with increased MEP amplitude (*p* < 0.05).

**Conclusion:**

The addition of iTBS or combined therapy to conventional rehabilitation improved UL motor function and activities of daily living (ADL) in patients with stroke. The iTBS group exhibited inhibitory effects on contralesional hemisphere excitability, while the MT group showed facilitative effects. These excitability changes were less pronounced in the combined treatment group.

**Clinical trial registration:**

Identifier ChiCTR1800015528.

## Highlights

- The trial examines the effectiveness of iTBS and MT in stroke patients, aiming to establish a well-developed method for addressing upper limb disability.
- This marks one of the initial clinical trials to assess the impacts of combining iTBS and MT on motor function among patients with stroke.
- Diverse outcome measures were used, encompassing motor function, daily activities, and cortical excitability.
- Patients underwent a brief (2 weeks) treatment period and were subsequently monitored for 3 months.
- The current sample volume is insufficient, and no imaging evaluation is performed. This electrophysiological assessment in the study only documented the changes prior to and following treatment, without providing long-term follow-up results.

## Introduction

Most individuals who have experienced a stroke suffer enduring harm, with nearly 80 percent developing lasting, work-constraining impairment in the upper limb (1, 2). Skillful control of the upper limb (UL) is vital for daily self-sufficiency and overall life satisfaction (3). Non-invasive brain stimulation (NIBS) is an approach used to activate specific brain regions, thereby modulating cortical excitability and function (4–6). Numerous inquiries have demonstrated positive therapeutic effects and their potential clinical significance in addressing the reestablishment of post-stroke interhemispheric imbalances (7–9). Research indicates that theta-burst stimulation (TBS) can activate the motor cortex by fostering long-term enhanced plasticity (10, 11). Different patterns of stimulation can elicit either stimulating [intermittent *θ* bursts (iTBS)] or inhibiting [continuous θ bursts (cTBS)] effects on brain excitability (12, 13). They observed that iTBS heightens motor-evoked potential (MEP) amplitudes, thus bolstering cerebral excitability, while cTBS dampens MEPs (14). Ackerley et al. identified that a 2-week application of iTBS to the ipsilesional M1 could potentially enhance precision grip-lift performance and Action Research Arm Test (ARAT) scores, which correlate with superior regulation of M1 corticospinal excitability (15, 16). Mirror therapy (MT) has been the focus of numerous investigations, with many studies highlighting its efficacy in enhancing grip strength (17), range of motion (18), movement speed (19), and manual dexterity (20) in stroke patients. Mirror visual feedback stimulates activity in several brain regions, primarily the primary motor cortex (M1) and the contralateral M1 (21). Prior research has indicated that separate application of MT and iTBS can effectively heighten cortical motor excitability in M1, contributing to the recovery of UL function (22, 23). But there are fewer reports on the effect of combination therapy for patients. In theory, the stimulating effect of using iTBS may make the brain state more receptive to the promoting effect of MT, thereby producing a stronger synergistic effect. This study aimed to investigate the impact of MT combined with iTBS on enhancing upper limb motor function and activities of daily living in patients with subacute stroke, and to further examine the influence of other factors on the efficacy of combined treatment. Neuroelectrophysiological techniques were employed to assess the impact of the treatment on cortical excitability.

## Methods and analysis

### Subjects

Stroke patients with upper limb impairment were enrolled from the Inpatient Rehabilitation Center of Tongji Hospital and Hubei Provincial Hospital of Integrated Chinese & Western Medicine. Enrollment followed the provision of informed consent for participation in this randomized double-blind controlled trial. Subjects were assigned randomly to one of four groups: control group (*N* = 17), MT group (*N* = 18), iTBS group (*N* = 18), and MT combined with iTBS group (*N* = 18). Measurements were taken on four occasions: at study commencement (T1; baseline), immediately following intervention (T2), 1 month post-intervention (T3), and 3 months post-intervention (T4). Assessments were conducted by two investigators who were unaware of the participants’ group assignments (refer to Table 1 for inclusion and exclusion criteria).

**Table 1:** Inclusion and exclusion criteria.

Criteria	Description
	All patients were diagnosed with stroke
Inclusion criteria	Patient age≥18 years;
	The first stroke occurred between 2 weeks and 6 month;
	Hand weakness (Brunnstrom 1–4); Motor evoked potential (MEP) can record from the unaffected hand.
	Cognitive clarity, Mini-mental State Examination (MMSE) > 21 (41);
	No visual perception disorder;
Exclusion criteria	History of epilepsy;
	Metallic implants in any part of body;
	Severe skull fracture, history of brain or spinal cord surgery (42);
	Administration of drugs that potentially lower seizure threshold (central nervous system stimulant, aminophylline, ephedrine);
	Significantly spasmodic (Ashworth score >2) (43);
	Aphasia or partial neglect;
	Malignant tumor, pregnancy;
	Serious heart, lung, liver, kidney and other diseases, inability to be followed up at regular interval;
	Enrolled otherclinical researches in 6 months before the trial.

### Patient and public involvement

In this study, a research consortium (comprising patient associations, nurses, and professional therapists) contributed to advising on study design, execution, and realization. A public symposium was organized to elucidate the benefits of MT and iTBS, stimulating active involvement among participants. The study schedule was designed to accommodate potential time conflicts with other therapies. Once subjects were randomized, every reasonable effort was made by the research consortium to ensure their participation for the entire study duration. Moreover, participants who enrolled in the program exempt from all evaluation and investigation costs associated with the study.

The risk of iTBS in patients with stroke include epilepsy, temporary hearing changes, and transient headache discomfort. To ensure treatment safety, patients with high-risk factors for epilepsy, such as a history of seizures or large cerebral infarcts, should be excluded. Most mild headaches are generally tolerable, while occasional persistent headaches can be managed with oral medication. All adverse events reported spontaneously by the subject to the investigator or his/her staff recorded for the period of the treatment (2 weeks). If a patient is unable to comply with the treatment protocol or experiences severe adverse reactions, the trial will be immediately discontinued.

Randomization was performed using computer-generated random numbers. After the blocks were numbered, a random number generator was used to select numbers according to the assigned subject sequence. Allocation information was sealed in opaque, numbered envelopes (24). In this double-blind trial, both participants and assessors were blinded to the nature of the treatment.

### Intervention

All patients received a standard rehabilitation regimen, which encompassed proper body positioning, active and passive activities for the hemiplegic limbs, balance training, and routine daily activities. For patients in the iTBS group, 600 pulses of excitatory head stimulation were administered once daily, 5 days a week, over a two-week span. A custom magnetic stimulator (YRD-CCI, Wuhan, China) and a figure-of-eight coil (6 cm in diameter with 3.5 T peak magnetic intensity) were used for delivering iTBS to the ipsilesional M1 by a trained investigator. A total of 600 pulses were delivered following the iTBS protocol (12). The stimulation site on the ipsilesional M1 was identified as the point of maximum MEP amplitude in the paretic Abductor Pollicis Brevis (APB) muscle, referred to as the “hotspot.” If MEP wasn’t evoked in the paralytic APB, iTBS was applied to the “hotspot” mirror site on the contralateral M1. The active motor threshold (AMT) was determined as subjects engaged in isometric contractions of the non-paralytic APB at around 20% of their maximum voluntary contraction, with more than 5 contractions in the contralateral APB muscle defined as indicative of a potential >200 μV in 10 trials. iTBS intensity was set at 80% of the AMT for the non-paralytic APB (25). Patients in the MT group underwent 30 min of MT training, consisting of 15 min of upper limb exercises (forearm pronation, wrist extension, finger extension, finger stretching, and specific isolated thumb and finger movements), followed by 15 min of task-oriented therapy (pouring water, drinking, writing, folding a towel, wiping a desk), guided by an experienced neurological physiotherapist. During the therapy, patients sat facing a mirror (60 × 90 cm) positioned perpendicular to their midline. The mirror obstructed the view of the affected hand, allowing the patient to observe the reflection of their healthy hand’s image. While moving the upper extremity, patients simultaneously observed the reflection of their unaffected limb in the mirror. With the assistance of an occupational therapist, the affected hand replicated the motion of the healthy side as closely as possible. In the combined group, iTBS was immediately followed by a 30-min session of personalized upper limb MT for 2 weeks.

Locate the Cz point (geometric center) of the patient.

HotSpot Target (M1) Selection for Motor Cortex Stimulation.

**Table:** 

Target Muscle	Standard Location	Alternative Landmark-Based Method
Left Hand	Contralateral C4 region	4–5 cm right lateral + 1–2 cm anterior to Cz
Right Hand	Contralateral C3 region	4–5 cm left lateral + 1–2 cm anterior to Cz

**Figure 1:**
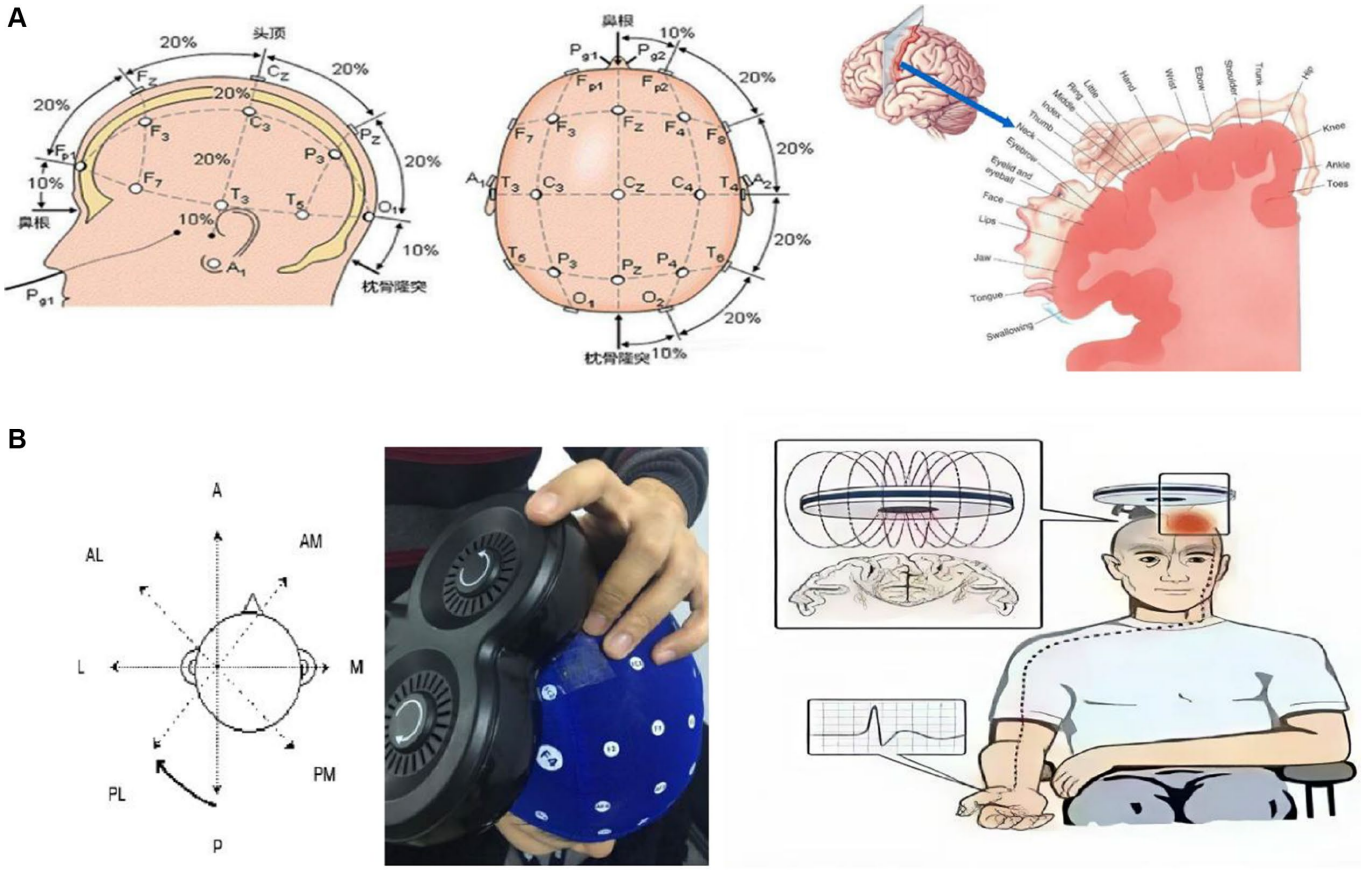
Study design and methods. **(A)** Methods for locating the M1 area of the head and its corresponding innervation regions; **(B)** Schematic diagrams of TBS treatment and MEP measurement.

Position the coil tangentially to the scalp, with the handle angled at 45 degrees to the sagittal plane. Place the electrodes over the abductor pollicis brevis muscle, following standard EMG protocols for motor evoked potential (MEP) recording. Electrode color coding (yellow/blue/black) conformed to international electrophysiological conventions (see Figure 1).

### Evaluation of the hypothesis

**Figure 2:**
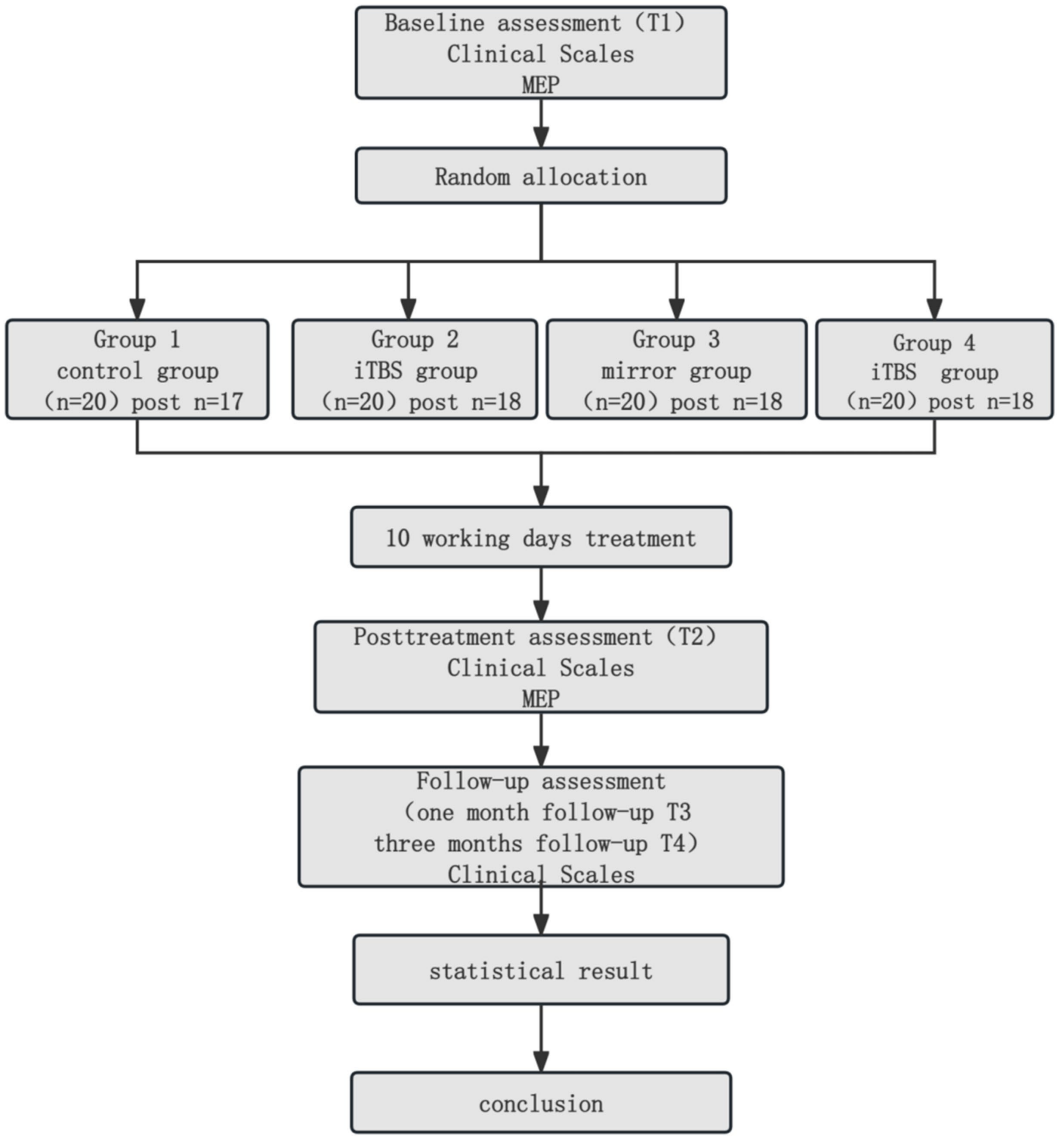
Schematic presentation of the experimental design.

We collected the following parameters from patients’ medical records at the outset of the trial (please refer to Table 2 and Figure 2 for the designated testing times). Stroke-related attributes encompassed stroke type (ischemic or hemorrhagic), disease duration, spasticity level, and neurological impairments.

**Table 2:** The designated testing times.

Instrument	T0	T1	T2	T3	T4
Inclusion/exclusion	X				
Demographics	X				
Stroke characteristics	X				
Sign informed consents	X				
Symptom assessment	X	X	X	X	X
NIHSS		X	X	X	X
FMA		X	X	X	X
MBI		X	X	X	X
SS-QOL		X	X	X	X
RMT、MEP		X	X		
Assessment of efficacy			X	X	X
Compliance evaluation		X	X		

(T1) occurred 1–7 days before intervention. Follow-up assessment was 1 day after treatment (T2), 1 month (T3) and 3 months after intervention (T4).

### Primary outcome measures

We utilized the Fugl-Meyer Assessment (FMA), a 33-item performance-based metric, to quantify upper limb motor function. Each item was rated on a three-tier ordinal scale (0 = incapable, 1 = partial capability, 2 = full capability), culminating in a maximum score of 66 points for upper limb motor performance (26, 27).

### Secondary outcome measures

We appraised the thresholds and amplitudes of bilateral MEPs as indicators of motor cortex excitability. We selected five waves with notable repeatability and significant amplitude (excluding the highest and lowest values) for calculating MEP latency and amplitude. RMT was defined as the minimum stimulus intensity needed to elicit a threshold MEP while at rest (50% uV of approximately 50 in 10 trials). All these assessments were conducted by a trained professional therapist.

The National Institutes of Health Stroke Scale (NIHSS) was employed to gauge stroke severity and disability at baseline (28). The Barthel Index (BI) measured activities of daily living (ADL) (29). The Stroke Specific Quality of Life Scale (SS-QOL) was administered to evaluate health status and overall quality of life (30).

### Data management

All statistical analyses were performed using the Statistical Package for Social Sciences (SPSS), v27.0. Descriptive analysis in SPSS depicted participant characteristics as mean and standard deviation. Changes before and after the two-week intervention were scrutinized for statistical significance through an ANOVA mixed model design, with Time (Baseline, Post, 1 Month, 3 Months) as a within-subject factor and Group (Basic, MT, iTBS, combined MT and iTBS) as a between-subject factor, encompassing FMS, NIHSS, BI, SS-QOL, RMT, and MEP. Paired t-tests with correction for multiple comparisons and LSD method were used for *post hoc* analysis. Normal distribution was verified with the Kolmogorov–Smirnov test prior to ANOVA data input. A significance level of p< 0.05 was employed for all tests.

## Results

**Figure 3:**
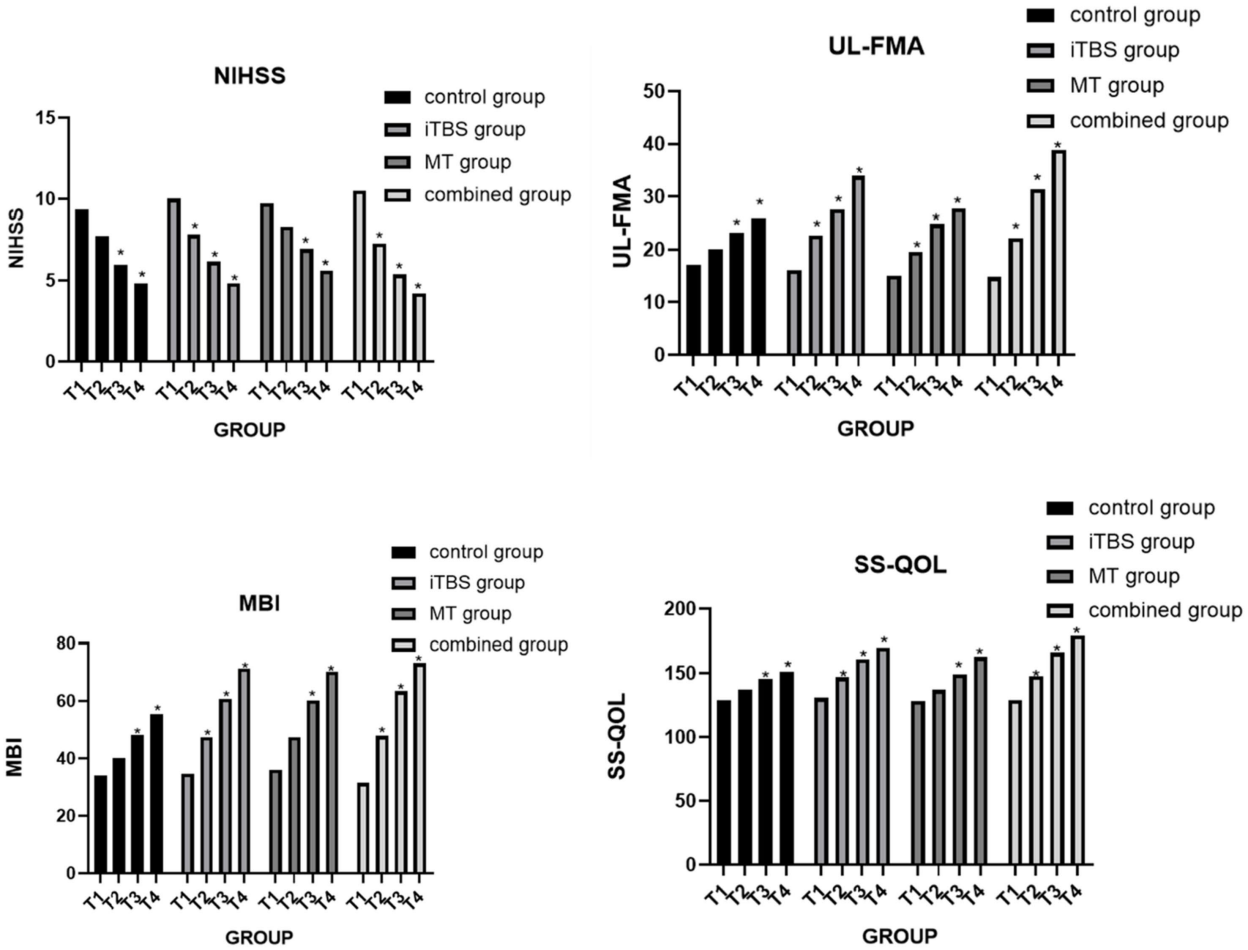
Clinical functional assessment. “*” indicates a statistically significant difference compared with the pre-treatment condition (*p* < 0.05). Post-treatment effects were statistically significant relative to baseline.

A total of 80 patients were randomly allocated into 4 groups, with 20 patients in each group. There were no significant statistical differences in baseline characteristics (including age, disease duration, gender ratio, and type of hemorrhage/infarction) among the groups. Furthermore, 9 patients were excluded from the study due to incomplete treatment cycles (two patients experienced headaches and insomnia following TBS treatment) or missing follow-up data (see Figure 3 and Table 3).

**Table 3:** General characteristics of the participants.

Characteristics of participants	control group (*n* = 17)	iTBS group (*n* = 18)	MT group (*n* = 18)	combined group (*n* = 18)	*F*	*p*
Age (year)	52.70 ± 14.71	53.38 ± 10.22	54.05 ± 9.61	50.33 ± 11.21	0.35	0.76
Sex (male/female)	11/6	11/7	12/6	10/8	–	0.909
Time since stroke (days)	59.52 ± 31.99	43.38 ± 16.96	51.50 ± 25.03	48.72 ± 27.05	1.19	0.31
Hemorrhagic/Ischemic	7/10	8/10	7/11	7/11	–	0.985
Paretic side (left/Right)	9/8	11/7	9/9	5/13	–	0.223
Sitting balance	2 (1, 3)	2 (1, 3)	2 (1, 3)	2 (1, 3)	–	0.994
Ashworth score	0 (0, 1)	0 (0, 1)	0 (0, 1)	0 (0, 2)	–	0.990

### Clinical evaluation results

**Figure 4:**
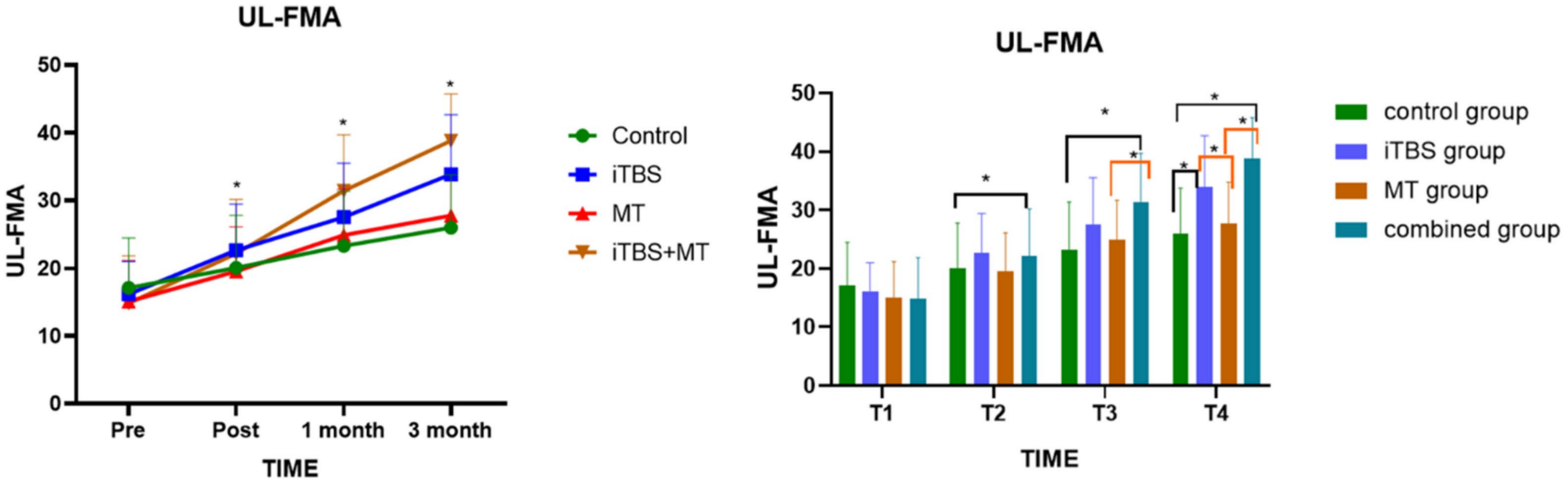
Effects of FMA score of upper limbs among groups at different time points. Symbol indicate mean values and error bars represent standard errors of the mean (Mean ± SD), * means compared with control group, *p < 0.05.

Before treatment, FMA scores showed no statistically significant differences among the four groups. However, intergroup comparisons revealed significant differences at treatment completion, 1 month post-treatment, and 3 months post-treatment. At the T2 time point, the combined treatment group showed a significant difference compared with the control group. At T3 and T4, the combined treatment group demonstrated significant differences relative to both the control and MT groups. Additionally, at T4, the iTBS group also showed significant differences compared with the control and MT groups (see Figure 4).

**Figure 5:**
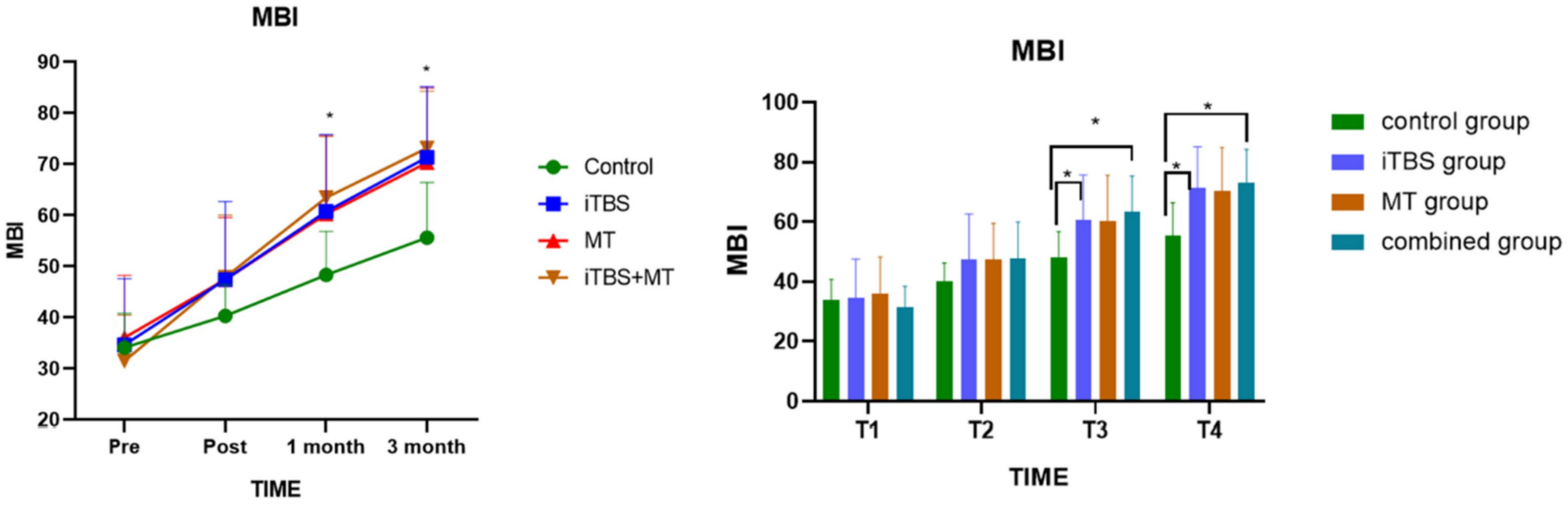
Effects of MBI score among groups at different time points; symbol indicate mean values and error bars represent standard errors of the mean (Mean ± SD), * means compared with control group, *p < 0.05.

Before treatment, MBI scores showed no statistically significant differences among the four groups. Intergroup comparisons revealed significant differences in MBI scores at 1 month and 3 months post-treatment. Further analysis showed that, at T3 and T4, the combined treatment group demonstrated significant differences compared with the control group. Additionally, the iTBS group exhibited a significant difference relative to the control group (see Figure 5).

**Figure 6:**
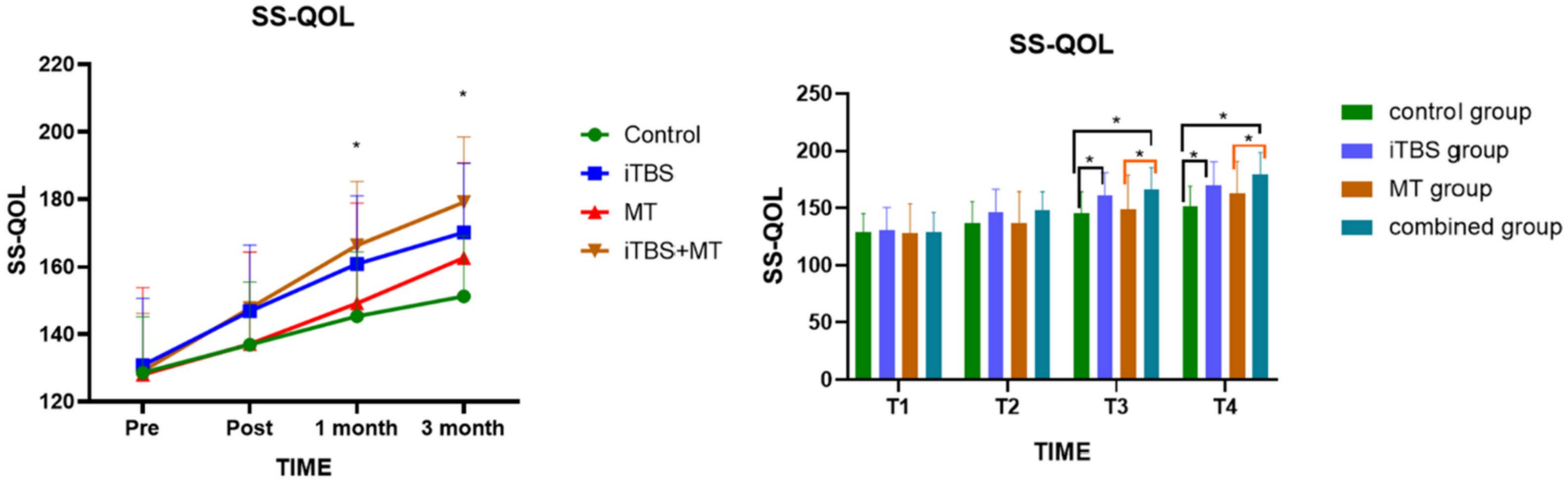
Effects of Stroke-specific quality of life scale (SS-QOL) among groups at different time points; symbol indicate mean values and error bars represent standard errors of the mean (Mean +/ - SD), * means compared with control group, *p < 0.05.

Before the intervention, SS-QOL scores showed no statistically significant differences among the four groups. Intergroup comparisons revealed significant differences at 1 month and 3 months post-treatment. Further analysis indicated that, at T3 and T4, the combined treatment group demonstrated significant differences compared with both the control and MT groups. Additionally, the iTBS group showed significant differences relative to the control group (see Figure 6).

### The RMT and MEP

Intra-group comparison of RMT, iTBS group increased, mirror treatment group decreased, there was statistical difference (*p* < 0.05). After 2 weeks of treatment, there was statistically significant difference between the four groups (*p* = 0.024). Further pairwise comparison, there was statistically significant difference between the iTBS group and the mirror treatment group after treatment (*p* = 0.014).

**Figure 7:**
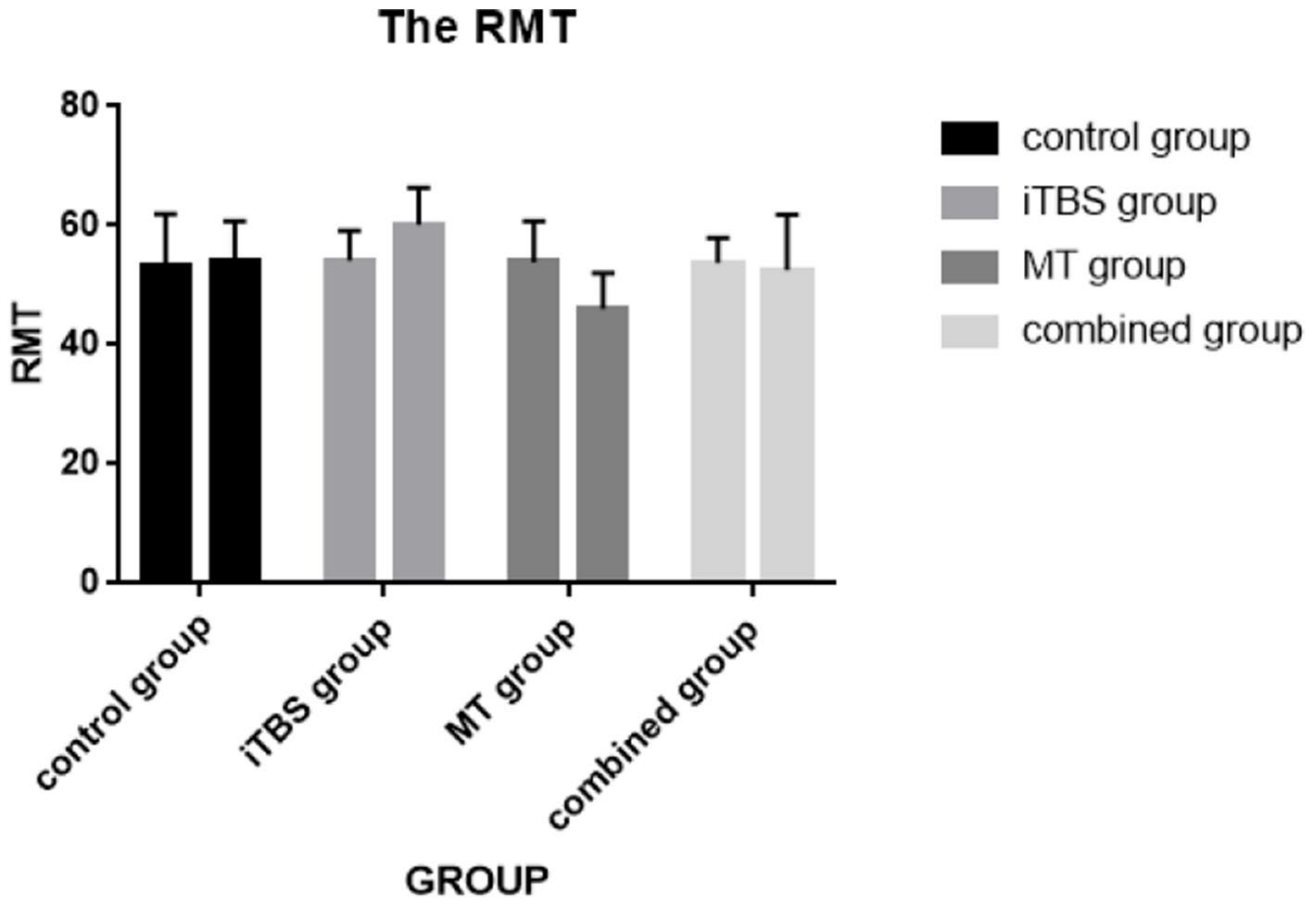
Intra-group and inter-group comparisons of RMT.

**Figure 8:**
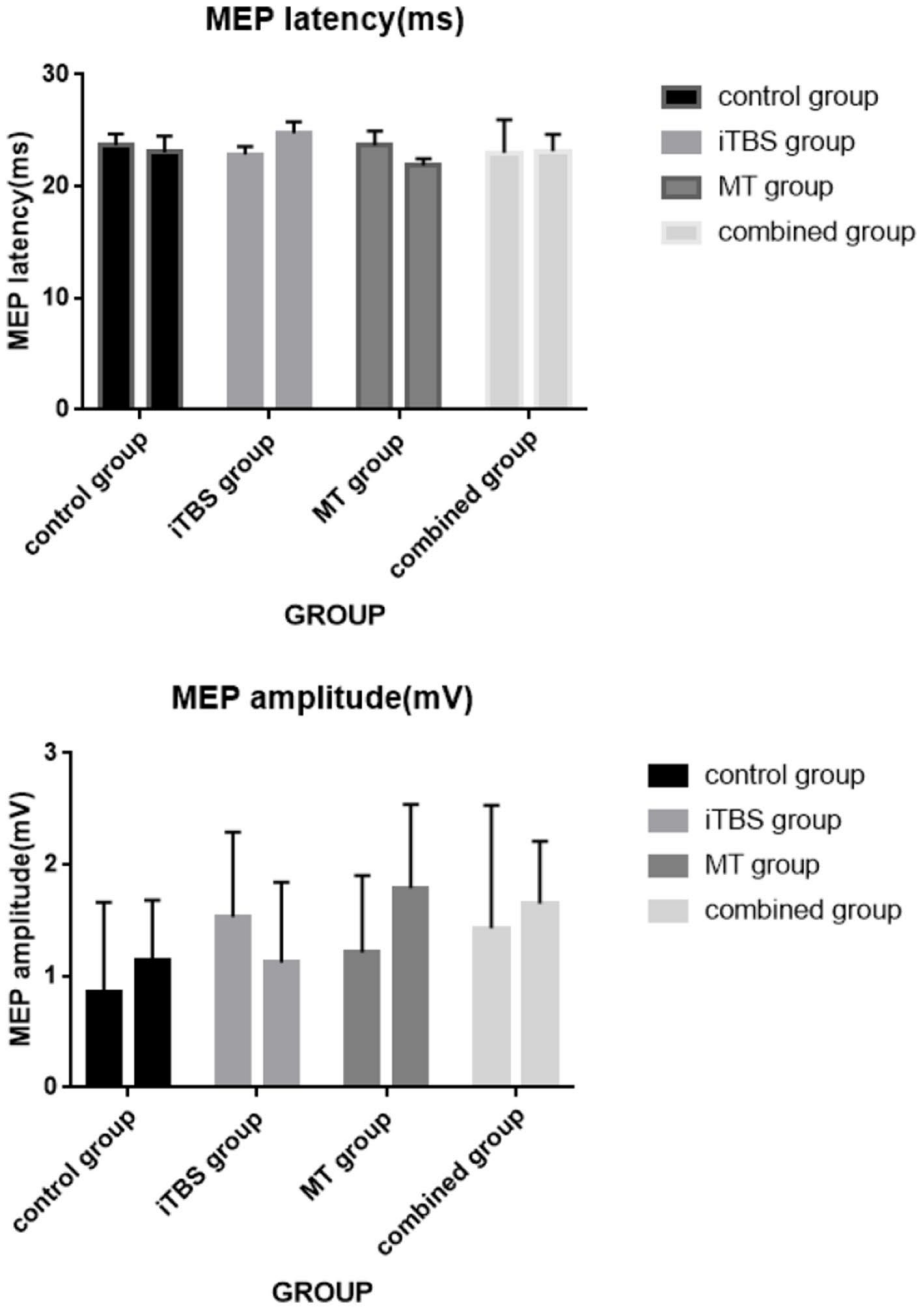
Intra-group and inter-group comparisons of MEP amplitude and MEP latency.

Intra-group comparison of LATENT MEP showed significant increase in iTBS group and decrease in mirror treatment group (*p* < 0.05). After 2 weeks of treatment, there was a statistical difference between the four groups (*p* = 0.005). Further pairwise comparison showed that there was a statistical difference between the iTBS group and the MT group after treatment (*p* = 0.002) (see Figures 7, 8).

There was statistical difference in MEP amplitude between iTBS and mirror group (*p* < 0.05). There was no significant difference between the four groups at each time point (*p* > 0.05).

## Discussion

This study demonstrated that combining iTBS with MT enhanced upper limb motor function and activities of daily living in patients with stroke. During the acute phase, motor cortex excitability on the affected side typically declined. As recovery progressed, neural activity in neighboring neurons near the lesion compensated for the damaged regions, reflecting the brain’s capacity for plasticity. Functional imaging and electrophysiological studies have supported the existence of this neuroplasticity. iTBS may promote cortical reorganization by enhancing cortical excitability, facilitating LTP-like plasticity (11), and modulating the responsiveness of M1 to input from other cortical areas. Notably, improvements in FMA scores at 1 month were associated with balanced corticomotor excitability and increased activation of the ipsilesional premotor cortex during grip tasks of the paretic hand. These effects likely contributed to improved planning, segmentation, and coordination of movements in the affected upper limb.

Imaging studies have shown increased activity in the perimotor cortex of the affected hemisphere during early stages of stroke recovery (31). Nojima et al. (32) confirmed, using transcranial magnetic stimulation, that motor function improvement following MT is more closely linked to remodeling within the primary motor cortex. During MT, consistent visual and somatosensory stimuli activate the mirror neuron system (MNS), induce neural remodeling (33), and facilitate recovery of upper motor function. In addition, MT involves bilateral upper limb training, performed either independently or with assistance. Symmetrical movements of both limbs result in widespread activation of motor cortical areas (34). It is hypothesized that mirror visual feedback may alleviate deficits in motor pathways on the affected side and promote upper limb motor recovery.

Facilitative TBS appeared to enhance the excitability of compensatory neurons on the affected side (35). Initiating iTBS demonstrated superior efficacy compared with sham stimulation in improving the therapeutic effects of robot-assisted training. Patients with relatively better upper limb function were more likely to benefit significantly from iTBS. This protocol was effectively used to enhance motor learning post-stroke (36). However, a study investigating the combined effects of iTBS (targeting the right dorsal premotor cortex, dPMC) and MT in healthy individuals over 4 days reported negative results (37). The findings suggested that iTBS increased excitability in left M1, which counteracted the effects of MT due to inhibitory interconnections between neural regions. Thus, applying iTBS to the right M1 in our study may have enhanced its responsiveness to stimulation, thereby improving function of the left upper limb compared with MT alone. TBS, a novel form of high-frequency repetitive rTMS delivered in bursts at low intensity, appeared safe and well tolerated.

Our results suggested that iTBS exerted an inhibitory effect on healthy cortex excitability in subacute stroke patients, implying facilitation of the affected cortex. Conversely, the MT group exhibited increased excitability in the healthy cortex, associated with bilateral M1 internal inhibition and reduced motor threshold (32, 38). Mechanistic studies of MT have shown that mirror visual feedback (MVF) reduces asymmetric hemispheric activation and supports cortical modifications in both ipsilateral and contralateral primary motor cortices (M1). Although MEPs from the affected side were elicited in only a few cases, an upward trend in cortical excitability was observed on the affected side. Studies indicate that during movement of the affected hand, activation shifts toward the affected hemisphere’s M1, reflecting enhanced activation on the affected side (39, 40). In the combined treatment group, no significant change in healthy cortex excitability was observed, possibly due to counteracting excitability interactions between the two hemispheres.

This study focused on refining strategies to address upper limb disability. However, the sample size was limited, and no imaging evaluation was conducted. The electrophysiological assessments documented changes only before and after treatment without long-term follow-up. Future multi-center studies with larger samples are needed to determine the optimal stimulation protocol to maximize therapeutic effects.

## Conclusion

To conclude, TBS exhibited a favorable inclination towards the enhancement of upper limb motor recovery in post-stroke patients. Moreover, the integration of iTBS and MT demonstrates a significant facilitatory effect on the recovery of upper limb motor function in stroke patients. The combined treatment approach exhibits superior efficacy compared to the use of either method in isolation. The underlying mechanism may involve the dual regulatory influence resulting from the interaction between the mirror neuron system and the cerebral cortex. Notwithstanding, limitations in this study include a limited sample size and the absence of neuroelectrophysiological findings 3 months after treatment. It is imperative that larger-scale, multicenter investigations be conducted to validate the effects of TBS on upper limb motor outcomes and cortical plasticity in stroke patients.

## Data Availability

The original contributions presented in the study are included in the article/Supplementary material, further inquiries can be directed to the corresponding authors.

## References

[1] Paul SL Srikanth VK Thrift AG. The large and growing burden of stroke. Curr Drug Targets. (2007) 8:786–93. doi: 10.2174/138945007781077418 17630931

[2] Langhorne P Coupar F Pollock A. Motor recovery after stroke: a systematic review. Lancet Neurol. (2009) 8:741–54. doi: 10.1016/S1474-4422(09)70150-4, PMID: 19608100

[3] Wang YC Magasi SR Bohannon RW Reuben DB McCreath HE Bubela DJ. Assessing dexterity function: a comparison of two alternatives for the NIH toolbox. J Hand Ther. (2011) 24:313–21. doi: 10.1016/j.jht.2011.05.001, PMID: 21798715 PMC3783205

[4] Lefaucheur JP André-Obadia N Antal A Ayache SS Baeken C Benninger DH. Evidence-based guidelines on the therapeutic use of repetitive transcranial magnetic stimulation (rTMS). Clin Neurophysiol. (2014) 125:2150–206. doi: 10.1016/j.clinph.2014.05.021, PMID: 25034472

[5] Simonetta-Moreau M. Non-invasive brain stimulation (NIBS) and motor recovery after stroke. Ann Phys Rehabil Med. (2014) 57:530–42. doi: 10.1016/j.rehab.2014.08.003, PMID: 25193774

[6] O'Brien AT Bertolucci F Torrealba-Acosta G Huerta R Fregni F Thibaut A. Non-invasive brain stimulation for fine motor improvement after stroke: a meta-analysis. Eur J Neurol. (2018) 25:1017–26. doi: 10.1111/ene.13643, PMID: 29744999

[7] Murase N Duque J Mazzocchio R Cohen LG. Influence of interhemispheric interactions on motor function in chronic stroke. Ann Neurol. (2004) 55:400–9. doi: 10.1002/ana.10848, PMID: 14991818

[8] Hsu WY Cheng CH Liao KK Lee I-H Lin Y-Y. Effects of repetitive transcranial magnetic stimulation on motor functions in patients with stroke: a meta-analysis. Stroke. (2012) 43:1849–57. doi: 10.1161/STROKEAHA.111.649756, PMID: 22713491

[9] Zhang L Xing G Fan Y Guo Z Chen H Mu Q. Short- and long-term effects of repetitive transcranial magnetic stimulation on upper limb motor function after stroke: a systematic review and Meta-analysis. Clin Rehabil. (2017) 31:1137–53. doi: 10.1177/0269215517692386, PMID: 28786336

[10] Huang YZ Rothwell JC Chen RS Lu CS Chuang WL. The theoretical model of theta burst form of repetitive transcranial magnetic stimulation. Clin Neurophysiol. (2011) 122:1011–8. doi: 10.1016/j.clinph.2010.08.016, PMID: 20869307 PMC3046904

[11] Huang YZ Chen RS Rothwell JC Wen H-Y. The after-effect of human theta burst stimulation is NMDA receptor dependent. Clin Neurophysiol. (2007) 118:1028–32. doi: 10.1016/j.clinph.2007.01.021, PMID: 17368094

[12] Huang YZ Edwards MJ Rounis E Bhatia KP Rothwell JC. Theta burst stimulation of the human motor cortex. Neuron. (2005) 45:201–6. doi: 10.1016/j.neuron.2004.12.033, PMID: 15664172

[13] Wischnewski M Schutter DJ. Efficacy and time course of theta burst stimulation in healthy humans. Brain Stimul. (2015) 8:685–92. doi: 10.1016/j.brs.2015.03.004, PMID: 26014214

[14] Talelli P Greenwood RJ Rothwell JC. Exploring Theta burst stimulation as an intervention to improve motor recovery in chronic stroke. Clin Neurophysiol. (2007) 118:333–42. doi: 10.1016/j.clinph.2006.10.014, PMID: 17166765

[15] Ackerley SJ Stinear CM Barber PA Byblow WD. Combining theta burst stimulation with training after subcortical stroke. Stroke. (2010) 41:1568–72. doi: 10.1161/STROKEAHA.110.583278, PMID: 20489170

[16] Ackerley SJ Stinear CM Barber PA Byblow WD. Priming sensorimotor cortex to enhance task-specific training after subcortical stroke. Clin Neurophysiol. (2014) 125:1451–8. doi: 10.1016/j.clinph.2013.11.020, PMID: 24360934

[17] Kim H Lee G Song C. Effect of functional electrical stimulation with mirror therapy on upper extremity motor function in poststroke patients. J Stroke Cerebrovasc Dis. (2014) 23:655–61. doi: 10.1016/j.jstrokecerebrovasdis.2013.06.017, PMID: 23867040

[18] Salhab G Sarraj AR Saleh S. Mirror therapy combined with functional electrical stimulation for rehabilitation of stroke survivors' ankle dorsiflexion. Conf Proc IEEE Eng Med Biol Soc. (2016) 2016:4699–702. doi: 10.1109/EMBC.2016.7591776, PMID: 28325013

[19] Lee D Lee G Jeong J. Mirror therapy with neuromuscular electrical stimulation for improving motor function of stroke survivors: a pilot randomized clinical study. Technol Health Care. (2016) 24:503–11. doi: 10.3233/THC-161144, PMID: 26890230

[20] Arya KN Pandian S Kumar D. Task-based mirror therapy enhances ipsilesional motor functions in stroke: a pilot study. J Bodyw Mov Ther. (2017) 21:334–41. doi: 10.1016/j.jbmt.2016.08.001, PMID: 28532877

[21] Saleh S Adamovich SV Tunik E. Mirrored feedback in chronic stroke: recruitment and effective connectivity of ipsilesional sensorimotor networks. Neurorehabil Neural Repair. (2014) 28:344–54. doi: 10.1177/1545968313513074, PMID: 24370569 PMC3989389

[22] Hsu YF Huang YZ Lin YY Tang CW Liao KK Lee PL. Intermittent theta burst stimulation over ipsilesional primary motor cortex of subacute ischemic stroke patients: a pilot study. Brain Stimul. (2013) 6:166–74. doi: 10.1016/j.brs.2012.04.007, PMID: 22659021

[23] Guo F Xu Q Abo Salem HM Yao Y Lou J Huang X. The neuronal correlates of mirror therapy: a functional magnetic resonance imaging study on mirror-induced visual illusions of ankle movements. Brain Res. (2016) 1639:186–93. doi: 10.1016/j.brainres.2016.03.002, PMID: 26972531

[24] Arya KN Pandian S Kumar D Puri V. Task-based Mirror therapy augmenting motor recovery in Poststroke hemiparesis: a randomized controlled trial. J Stroke Cerebrovasc Dis. (2015) 24:1738–48. doi: 10.1016/j.jstrokecerebrovasdis.2015.03.026, PMID: 26096318

[25] Iezzi E Conte A Suppa A Agostino R Dinapoli L Scontrini A. Phasic voluntary movements reverse the aftereffects of subsequent theta-burst stimulation in humans. J Neurophysiol. (2008) 100:2070–6. doi: 10.1152/jn.90521.2008, PMID: 18753328

[26] Badke MB Duncan PW. Patterns of rapid motor responses during postural adjustments when standing in healthy subjects and hemiplegic patients. Phys Ther. (1983) 63:13–20. doi: 10.1093/ptj/63.1.13, PMID: 6849002

[27] Gladstone DJ Danells CJ Black SE. The fugl-meyer assessment of motor recovery after stroke: a critical review of its measurement properties. Neurorehabil Neural Repair. (2002) 16:232–40. doi: 10.1177/154596802401105171, PMID: 12234086

[28] Hinkle JL. Reliability and validity of the National Institutes of Health stroke scale for neuroscience nurses. Stroke. (2014) 45:e32–4. doi: 10.1161/STROKEAHA.113.004243, PMID: 24496393

[29] Cid-Ruzafa J Damian-Moreno J. Disability evaluation: Barthel's index. Rev Esp Salud Publica. (1997) 71:127–37. 9546856

[30] Wong GK Lam SW Ngai K Wong A Poon WS Mok V. Development of a short form of stroke-specific quality of life scale for patients after aneurysmal subarachnoid hemorrhage. J Neurol Sci. (2013) 335:204–9. doi: 10.1016/j.jns.2013.09.033, PMID: 24120271

[31] Rossini PM Calautti C Pauri F Baron JC. Post-stroke plastic reorganisation in the adult brain. Lancet Neurol. (2003) 2:493–502. doi: 10.1016/S1474-4422(03)00485-X, PMID: 12878437

[32] Nojima I Mima T Koganemaru S Thabit MN Fukuyama H Kawamata T. Human motor plasticity induced by mirror visual feedback. J Neurosci. (2012) 32:1293–300. doi: 10.1523/JNEUROSCI.5364-11.2012, PMID: 22279214 PMC6796271

[33] Garrison KA Winstein CJ Aziz-Zadeh L. The mirror neuron system: a neural substrate for methods in stroke rehabilitation. Neurorehabil Neural Repair. (2010) 24:404–12. doi: 10.1177/1545968309354536, PMID: 20207851 PMC11692383

[34] Neva JL Vesia M Singh AM Staines WR. Modulation of left primary motor cortex excitability after bimanual training and intermittent theta burst stimulation to left dorsal premotor cortex. Behav Brain Res. (2014) 261:289–96. doi: 10.1016/j.bbr.2013.12.029, PMID: 24388976

[35] He W Au-Yeung SY Mak M Au-Yeung S-y S Leung TWH Leung H. The potential synergism by combining external counterpulsation with intermittent theta burst stimulation in post-stroke motor function recovery. Med Hypotheses. (2016) 93:140–2. doi: 10.1016/j.mehy.2016.05.024, PMID: 27372874

[36] Zhang JJ Bai Z Fong KNK. Priming intermittent Theta burst stimulation for Hemiparetic upper limb after stroke: a randomized controlled trial. Stroke. (2022) 53:2171–81. doi: 10.1161/STROKEAHA.121.037870, PMID: 35317611

[37] Läppchen CH Ringer T Blessin J Schulz K Seidel G Lange R. Daily iTBS worsens hand motor training—a combined TMS, fMRI and mirror training study. NeuroImage. (2015) 107:257–65. doi: 10.1016/j.neuroimage.2014.12.022, PMID: 25514515

[38] Läppchen CH Ringer T Blessin J Seidel G Grieshammer S Lange R. Optical illusion alters M1 excitability after mirror therapy: a TMS study. J Neurophysiol. (2012) 108:2857–61. doi: 10.1152/jn.00321.2012, PMID: 22972955

[39] Bhasin A Padma Srivastava MV Kumaran SS Bhatia R Mohanty S. Neural interface of mirror therapy in chronic stroke patients: a functional magnetic resonance imaging study. Neurol India. (2012) 60:570–6. doi: 10.4103/0028-3886.105188, PMID: 23287316

[40] Michielsen ME Selles RW Van Der Geest JN Eckhardt M Yavuzer G Stam HJ. Motor recovery and cortical reorganization after mirror therapy in chronic stroke patients: a phase II randomized controlled trial. Neurorehabil Neural Repair. (2011) 25:223–33. doi: 10.1177/1545968310385127, PMID: 21051765

[41] Babacan-Yildiz G Ur-Ozcelik E Kolukisa M Turan Işik A Gürsoy E Kocaman G. Validity and reliability studies of modified Mini mental state examination (MMSE-E) for Turkish illiterate patients with diagnosis of Alzheimer disease. Turk Psikiyatri Derg. (2016) 27:41–6. 27369684

[42] Rossi S Hallett M Rossini PM Pascual-Leone A Safety of TMS Consensus Group. Safety, ethical considerations, and application guidelines for the use of transcranial magnetic stimulation in clinical practice and research. Clin Neurophysiol. (2009) 120:2008–39. doi: 10.1016/j.clinph.2009.08.016, PMID: 19833552 PMC3260536

[43] Akpinar P Atici A Ozkan FU Aktas I Kulcu DG Sarı A. Reliability of the modified Ashworth scale and modified Tardieu scale in patients with spinal cord injuries. Spinal Cord. (2017) 55:944–9. doi: 10.1038/sc.2017.48, PMID: 28485384

