# Supplementary material for: Effects of mirror therapy combined with theta burst stimulation on motor recovery of upper limbs after stroke: a randomized controlled study
Source: Front Neurol. 2025 Jul 11;16:1548703. doi: 10.3389/fneur.2025.1548703 (PMC12292015; doi:10.3389/fneur.2025.1548703)
Supplement: Supplementary file 1 [file Table_1.doc]

|  | n | NIHSS | FMA | MBI | SS-QOL |
| --- | --- | --- | --- | --- | --- |
| control group | | | | |  |
| T1 | 17 | 9.35±3.23 | 17.11±7.35 | 34.05±6.71 | 128.58±16.58 |
| T2 | 17 | 7.76±2.81 | 20.05±7.74 | 40.29±6.04 | 136.88±18.65 |
| T3 | 17 | 5.94±2.65^a^ | 23.29±8.06^a^ | 48.29±8.53^a^ | 145.35±19.02^a^ |
| T4 | 17 | 4.82±2.12^a^ | 26.00±7.78^a^ | 55.58±10.81^a^ | 151.23±17.89^a^ |
| iTBS group | | | | |  |
| T1 | 18 | 10.00±3.62 | 16.16±4.84 | 34.61±12.95 | 130.77±19.85 |
| T2 | 18 | 7.83±3.27^a^ | 22.66±6.79^a^ | 47.38±15.27^a^ | 146.83±19.58^a^ |
| T3 | 18 | 6.16±2.93^a^ | 27.55±7.98^a^ | 60.72±15.01^ab^ | 160.83±20.17^ab^ |
| T4 | 18 | 4.83±2.47^a^ | 33.88±8.79^abc^ | 71.33±13.82^ab^ | 170.16±20.46^ab^ |
| MT group | | | | |  |
| T1 | 18 | 9.72±2.86 | 15.11±6.07 | 36.00±12.21 | 128.00±25.75 |
| T2 | 18 | 8.33±2.70 | 19.55±6.57^a^ | 47.38±12.16 | 137.11±27.31 |
| T3 | 18 | 6.94±2.81^a^ | 24.88±6.82^a^ | 60.22±15.41^ab^ | 149.16±29.70^a^ |
| T4 | 18 | 5.61±2.52^a^ | 27.77±7.00^a^ | 70.27±14.59^ab^ | 162.66±28.13^a^ |
| combined group | | | | |  |
| T1 | 18 | 10.50±2.81 | 14.83±7.01 | 31.44±8.99 | 129.11±17.05 |
| T2 | 18 | 7.27±2.90^a^ | 22.16±8.01^ab^ | 47.83±12.16^a^ | 147.72±16.57^a^ |
| T3 | 18 | 5.38±2.40^a^ | 31.44±8.25^abc^ | 63.44±11.95^ab^ | 166.33±18.96^abc^ |
| T4 | 18 | 4.22±2.15^a^ | 38.83±6.92^abc^ | 73.11±11.16^ab^ | 179.11±19.30^abc^ |
